# The metabolic repression effect of carbon-ion radiotherapy in synchronous hormone-sensitive oligometastatic prostate cancer

**DOI:** 10.3389/fendo.2023.1291653

**Published:** 2023-11-13

**Authors:** Zhenshan Zhang, Yulei Pei, Wei Hu, Yushan Xue, Renli Ning, Xiaomao Guo, Yun Sun, Qing Zhang

**Affiliations:** ^1^ Department of Radiation Oncology, Shanghai Proton and Heavy Ion Center, Fudan University Cancer Hospital, Shanghai, China; ^2^ Shanghai Key Laboratory of Radiation Oncology, Shanghai, China; ^3^ Shanghai Engineering Research Center of Proton and Heavy Ion Radiation Therapy, Shanghai, China; ^4^ Department of Research and Development, Shanghai Proton and Heavy Ion Center, Fudan University Cancer Hospital, Shanghai, China

**Keywords:** carbon ion radiotherapy, metastatic prostate cancer, metabolomics, hormone treatment, glutamine

## Abstract

**Background:**

Metastatic prostate cancer (PCa) poses a significant public health concern. While radiation therapy (RT) is commonly utilized in the treatment of synchronous oligometastatic hormone sensitive prostate cancer (OM-HSPC), the occurrence of biochemical recurrence still remains. To deepen our understanding and optimize the outcome of OM-HSPC, we conducted this study to investigate the characteristics of PCa progression and explore potential synergistic mechanisms involving carbon-ion radiotherapy (CIRT) and neoadjuvant androgen deprivation treatment (naADT) in OM-HSPC.

**Methods:**

Metabolomic analysis was conducted with 72 urinary samples (at different timepoints) from 33 Patients (T2-3N0M0-1b) and 18 healthy volunteers by using liquid chromatography-tandem mass spectrometry (LC-MS/MS). MetaboAnalyst website and R software were employed for metabolomic analysis and visualization (using the criteria of *p* value < 0.05 and |FC|>1.5). The impact of CIRT on metabolism were further verified and explored through *in vitro* and *in vivo* experiments.

**Results:**

We found that most metabolites (223 out of 233) were upregulated in treatment-naïve PCa samples compared to healthy samples. After naADT, 60 core risk metabolites were still significantly related to PCa’s progression, and the glutamine level which was significantly higher in OM-HSPC compared to other groups. Remarkably, after CIRT treatment, the glutamine levels in OM-HSPC were significantly reduced to the level of healthy samples. Experiments further confirmed CIRT’s ability to suppress glutamine levels in PCa tumors and its potential enhancement with glutamine deprivation intervention.

**Conclusion:**

CIRT with naADT might synergistically inhibit HS-OMPC development, progression and even the ADT resistance through glutamine metabolism repression, moreover, the glutamine metabolism might be a novel target to further improved the efficacy of CIRT.

## Introduction

Approximately 15% of prostate cancer (PCa) cases were metastatic at diagnosis, with a 5-year survival rate of 31% (1). There is a growing public health emphasis on optimizing the treatment strategies for oligometastatic hormone-sensitive prostate cancer (OM-HSPC) to improve patient prognosis. With photon combined with androgen deprivation treatment (ADT) being a standard of care, the median overall survival (OS) was 41.9 months in the STAMPED trial (2) and 47 months in the HORRAD trial (3) for OM-HSPC patients. The quest to further enhance these outcomes remains a significant concern for radiation oncologists.

Carbon ion radiotherapy (CIRT) as a relatively new innovation of irradiation has different killing mechanisms and radiobiological effects than photon irradiation. Numerous studies (4–7) reported that CIRT could improve the efficacy and lower the toxicities for localized prostate cancer compared to conventional photon radiotherapy. In 2021, we reported the first study of CIRT-induced metabolic reprogramming in PCa and explored a metabolic PET/CT imaging-guided simultaneous integrated boost (SIB) CIRT technique and further inhibited localized PCa metabolism, which realized global metabolite level inhibition (8, 9). Moreover, whether the biomarkers in photon radiotherapy still work in CIRT is unclear, and for CIRT, the current biomarkers, such as IGFBP-3 (10), are still insufficient and needed further clinical confirmation. We hypothesized CIRT could also reflect its efficacy and characteristics in OM-HSPC treatment via metabolism remodeling.

In this study, we analyzed the metabolic profiles of varying stages and statuses of PCa—including healthy individuals, intermediate-risk PCa, high-risk PCa, and OM-HSPC—using untargeted metabolomics to pinpoint risk compounds. Given the challenges in obtaining PCa tissue from patients, we pursued both *in vivo* and *in vitro* experiments to discern the genuine state of key risk metabolites in tumor tissue or PCa cells post-CIRT. Additionally, we deprived the glutamine in media, aiming to further inhibit tumor cells’ invasion and migration competence. Our results shed light on the metabolic mechanisms underpinning the combination of CIRT and naADT and the treatment responses across different PCa stages to naADT. Remarkably, this research unveils, for the first time, the biomarker and target value of glutamine in PCa CIRT.

## Materials and methods

### Patients and staging evaluation

In this study, 33 patients were involved who had pathologically confirmed prostate adenocarcinoma staged as T2-3N0M0-1b based on the 2008 AJCC/UICC staging classification. Additionally, 18 healthy volunteers, who were within a similar age range as the patients, participated. Consequently, we successfully collected a total of 72 urinary samples. These samples were categorized into four groups:

1. Healthy Group: This consisted of 18 samples, each from a different healthy volunteer.
2. Treatment-Naïve Group: This group comprised 18 samples from 18 distinct patients (details in **Table 1**).
3. Pre-CIRT Group: 15 samples derived from 15 unique patients, and 3 samples form 3 patients overlapped with the treatment-naïve group, this group had 18 samples (details in **Table 2**).
4. Post-CIRT Group: Using the same patients from the pre-CIRT.

**Table 1:** Clinical information of treatment-naïve group.

Characteristics	Overall
Age, n (%)	
<60	1 (5.6%)
≥60 and <70	6 (33.3%)
≥70 and <80	11 (61.1%)
T, n (%)	
T2	13 (72.2%)
T3	4 (22.2%)
T4	1 (5.6%)
M, n (%)	
M0	15 (83.3%)
M1b	3 (16.7%)
Risk group, n (%)	
I	3 (16.7%)
II	8 (44.4%)
III	3 (16.7%)
IV	4 (22.2%)
Gleason score, n (%)	
6	4 (22.2%)
7	9 (50%)
≥8	5 (27.8%)
Highest PSA (ng/ml), median (IQR)	10.65 (6.285, 14.435)

**Table 2:** Clinical information of pre-CIRT and post-CIRT group.

Characteristics	OM-HSPC	Intermediate Risk	High Risk	*p* value
n	6	6	6	
Age, n (%)				0.175
≥60 and <70	2 (33.3%)	3 (50%)	2 (33.3%)	
≥70 and <80	4 (66.7%)	3 (50%)	1 (16.7%)	
≥80	0 (0%)	0 (0%)	3 (50%)	
T, n (%)				0.149
T2	3 (50%)	6 (100%)	4 (66.7%)	
T3	3 (50%)	0 (0%)	1 (16.7%)	
Tx	0 (0%)	0 (0%)	1 (16.7%)	
Pre-CIRT testosterone (nmol/l), median (IQR)	0.13 (0.13, 0.15)	0.21 (0.19, 0.30)	0.16 (0.15, 0.23)	0.153
Pre-CIRT PSA (ng/ml), mean ± sd	0.08 ± 0.09	0.41 ± 0.42	0.43 ± 0.48	0.208
Gleason score, n (%)				0.128
6	1 (16.7%)	1 (16.7%)	1 (16.7%)	
7	1 (16.7%)	5 (83.3%)	3 (50%)	
≥8	4 (66.7%)	0 (0%)	2 (33.3%)	
Month after naADT, median (IQR)	4.72 (4.22, 10.36)	1.98 (1.89, 2.98)	3.00 (2.15, 3.8)	**0.019**

Risk groups were identified according to the National Comprehensive Cancer Network (NCCN) guidelines. OM-HSPC, oligo-metastatic hormone sensitive prostate cancer (synchronous, number of bone metastasis lesion ≤ 5). Bold values means the P value was less than 0.05.

Synchronous OM-HSPC was defined as the number of bone metastasis lesions detected on both PSMA PET/CT and MRI imaging ≤ 5, without visceral/other metastasis. The criteria patients’ selection was performed according to the following criteria.

1. Patients were excluded if they had other uncontrolled primary malignancies, lymph nodes or distant metastasis, previous prostatectomy or pelvic radiotherapy, drug abuse or alcohol dependence, previous use of immunosuppressive therapies, or had infectious or metabolic disease, HIV or hepatitis virus, syphilis, common cold, diabetes and hyperuricemia.
2. Samples were successfully collected and processed within 4 h.
3. The level of testosterone pre-CIRT should be under 1.7 nmol/L.

### CIRT

CIRT was administered using the Siemens IONTRIS particle therapy system. The delineation of the clinical target volume (CTV) was determined based on the ESTRO ACROP consensus (11). Depending on the different risk groups, the CTV for CIRT comprised the entire prostate gland and part of the seminal vesicles. For intermediate-risk patients, the CTV included the complete prostate gland and the lower 1-1.5 cm of the seminal vesicles, and for high-risk patients, the CTV encompassed the entire prostate gland and the lower 2-2.5 cm of the seminal vesicles. For patients with synchronous OM-HSPC, the CTV included the entire prostate gland and entire seminal vesicle and the positive bone lesion(s) identified on both PSMA PET/CT and MRI imaging.

CIRT was carried out using the Siemens IONTRIS particle therapy system. The prescribed dose was determined using the Syngo planning system (V13B, Siemens, Germany), taking into account the local effect model (LEM) 1. For intermediate- and high-risk patients, the CIRT dose was 65.6 Gy (RBE) at 4.1Gy(RBE) per daily fraction. For OM-HSPC patients, the CIRT doses was 65.6 Gy (RBE) at 4.1Gy(RBE) per daily fraction to the whole prostate and the positive bone lesion(s) was treated using metastasis-directed therapy (MDT) with 49.2 Gy (RBE) at 4.1Gy(RBE) per daily fraction. 100% of the CTV was prescribed to be covered by 95% isodose line, while 100% of the planning target volume (PTV) was prescribed to be covered by 90% isodose line. In order to ensure accurate treatment delivery, both bladder and rectum preparation were performed before each treatment.

### Collection and preparation of urinary samples

Urine samples were collected from the patients within a time frame of 4 hours before the administration of the initial fraction and within 4 hours after the completion of the final fraction. Following collection, the samples were promptly stored at a temperature of 4°C. To eliminate potential bacterial contamination, a 0.22 µm membrane filter was utilized. In each thawed sample, 80 μL of urine was combined with 160 μL of acetonitrile/methanol (1:1, v/v) in a 1.5 mL tube and placed in an ice bath. After vortexing for a duration of 30 seconds, the mixture underwent further treatment through ultrasonication for 10 minutes while placed in an ice/water bath. Subsequently, the tube was stored at a temperature of -20°C for at least 1 hour. After centrifugation (12000 rpm, 15 minutes) at 4°C, 200 μL of supernatant was transferred to a sample vial insert (250 μL) at 4°C, awaiting injection into LC-MS. Simultaneously, a pooled quality control (QC) sample was prepared by combining equal volumes (5 µL) of the supernatant from each sample.

### High-throughput UPLC-MS/MS analysis

Ultrapure water was acquired from the Millipore Milli-Q Integral 5 Ultrapure Water Systems (Billerica, USA). LC-MS grade acetonitrile and methanol were procured from SCRC (Shanghai, China). Ammonium hydroxide and ammonium formate were purchased from Merck (Darmstadt, Germany). All standard compounds were obtained from Tansoole (Shanghai, China), ThermoFisher (Waltham, USA), Merck, Aladdin (Shanghai, China), and Macklin (Shanghai, China).

The UPLC system (ExionLC™ 2.0, AB SCIEX, USA) coupled with a quadruple time-of-flight mass spectrometer (X500B, AB SCIEX, USA) was used to collect the metabolomics data of PBMC samples. Metabolite separation in both positive and negative modes was performed using an ACQUITY UPLC BEH Amide Column, 130 Å, 1.7 µm, 2.1 mm × 100 mm (Waters, USA). The water phase (A) was prepared by mixing 25 mmol of ammonium hydroxide and 25 mmol of ammonium formate with 1 L of water, while the organic phase (B) consisted of acetonitrile. The total liquid flow rate was set at 0.3 mL/min, and the gradient elution program was as follows: 0–1 min: 95% B, 1–14 min: 95% B to 65% B, 14–16 min: 65% B to 40% B, 16–18 min: 40% B, 18–18.1 min: 40% B to 95% B, 18.1–23 min: 95% B. The injection volume was 2 μL, and the column oven temperature was maintained at 40 °C.

During the analysis queue, all samples were randomly injected. Mass axis calibration, blank sample injection, pooled QC injection, and laboratory mixed standard compound solution injection were performed sequentially every eight sample injections. For electrospray ionization source parameters, ion source gas 1 and ion source gas 2 were set at 55 psi, curtain gas at 35 psi, temperature at 600 °C, and spray voltage at 5500 V or -4500 V in positive or negative modes, respectively. The TOF MS scan parameters included a mass scan range of 50–1000 Da, an accumulation time of 0.2 s, a declustering potential of 60 V or -60 V in positive or negative modes, respectively, and a collision energy of 10 V or -10 V in positive or negative modes, respectively. The acquisition of MS/MS spectra was triggered using the information-dependent acquisition mode with a maximum of ten candidate ions, and dynamic background subtraction was enabled. For TOF MS/MS scan parameters, the mass scan range was set as 25–1000 Da, the accumulation time as 0.05 s, the collision energy as 35 V or -35 V in positive or negative modes, respectively, and the collision energy spread as 15. The default settings were retained for other items.

The raw data files (.wiff2 and.wiff.scan) were converted to mzXML format and mgf format using ProteoWizard with the Peak Picking filter to centroid the peaks. The relative quantitative matrixes of all samples were generated using our large-scale metabolomics data processing software based on the R language (Software copyright registration Number: 2023SR0256527). Initially, all metabolite peaks were annotated using MetDNA2 (http://metdna.zhulab.cn/). Subsequently, the statistical significance of unknown peaks was further annotated with available reference standards in our laboratory and web-based resources, including the Human Metabolome Database (HMDB) (http://www.hmdb.ca/), Massbank (http://www.massbank.jp), and PubChem (pubchem.ncbi.nlm.nih.gov).

### Glutamine-associated gene set and public RNA-seq data acquisition

14 gene sets of glutamine metabolism were obtained from MSigDB (https://www.gsea-msigdb.org/gsea/msigdb) (12, 13) via inputting “glutamine” to search blank. After the removal of duplicate genes, a gene set consisting of 128 genes was established.

The RNA-seq data and corresponding clinical information of the Cancer Genome Atlas (TCGA) (14) prostate cancer cohorts were acquired from the UCSC (http://xena.ucsc.edu/) (15) public database.

### Cell and mouse

PC3 cells were obtained from the Chinese Academy of Sciences Cell Bank and cultured in 1640 medium (Gibco Thermo Fisher Scientific, Inc. USA) supplemented with 10% fetal calf serum (FBS) (Gibco Thermo Fisher Scientific, Inc. USA), 100 U/mL penicillin, and 100 μg/mL streptomycin (Solarbio, Beijing, China) and cultured at 37°C in a 5% CO_2_ humid atmosphere. Four groups of cells were designed in each *in vitro* experiment: negative control (NC) with complete medium [NC gln(+)], NC with glutamine-free medium [NC gln(-)], CIRT with completed medium [CIRT gln(+)] and CIRT with glutamine-free medium [CIRT gln(-)].

Mice were obtained from Hangzhou Ziyuan Laboratory Animal Technology Co., Ltd. The animal ethics committee of Shanghai Proton and Heavy Ion Center approved the use of mice for experiments.

### Tumor growth assay and related preparation of metabolomic analysis

Male BALB/c-nude mice aged 6 weeks were obtained, and PC3 cells were suspended in the culture medium at a concentration of 5 × 10^6^ cells/mL. A 200 μL cell suspension was then subcutaneously injected into the right flank of 10 nude mice. The volume of tumors was measured using the following formula: length × width^2^ × 0.5 (mm³). Irradiation commenced on day 10 after tumor inoculation when the mean tumor volume reached approximately 100 mm³. Euthanasia was carried out by exposing the mice to carbon dioxide. The percentage of chamber volume replaced by carbon dioxide flow was approximately 50% vol/min.

Harvested tumor samples were utilized for further metabolomic analysis, and the steps were as follows: approximately 12 mg (record accurate weight) solid tissue was added to methanol/water (1:1, 480 μL solution per 12 mg tissue) in an ice bath. After homogenization with an automatic tissue separator or other methods, 480 μL homogenate was added to 720 μL acetonitrile/methanol (2:1). After vortexing for 30 s, the mixture was further treated with ultrasonication for 10 min in an ice/water bath. Then, this tube was refrigerated at −20 °C for at least 1 h. After centrifugation (12000 rpm, 15 min) at 4 °C, 1 mL of supernatant was transferred to a new tube. Then, all solvent was removed by a vacuum concentrator at room temperature for 3 h or longer. The obtained metabolite solid could be stored at −80 °C until testing. Before testing with LC-MS, the metabolite solid was redissolved in 100 μL acetonitrile/water (1:1). After vortexing for 30 s, the mixture was further treated with ultrasonication for 5 min in an ice/water bath. Then, the mixture was centrifuged (12000 rpm, 15 min) at 4 °C. The obtained solution was stored in sample vial inserts (250 μL) at 4 °C until LC-MS injection.

### Transwell assay

Transwell assay using 24-well plates with an 8-µm pore chamber (Corning, Inc.), and Matrigel matrix was diluted 1:8 to coat the upper side of the membrane at the bottom of the Transwell chamber. Cells were added to the upper chamber (3 × 10^4 cells/well). Meanwhile, 1640 medium with 10% FBS was added to the lower compartment. Then, the plates were incubated for 48 h in the incubator. After incubation, cells that migrated to the lower surface of the filter membrane were fixed with 4% paraformaldehyde and stained with 0.5% crystal violet. Cells remaining on the upper surface of the filter membrane were gently scraped with a cotton swab. The lower surfaces were captured by an inverted microscope, and the counting was repeated three times.

### Wound healing assay

PC3 cells were seeded into a 6-well plate. When the cell confluence reached 80%, we scratched the monolayer cells with a sterile micropipette tip. The floating cells were then washed with PBS. Wound healing within the scrape line was observed at 0 h, 16 h and 24 h. Triplicate wells for each condition were examined.

### Statistics

The data analysis was conducted using MetaboAnalyst (https://metaboanalyst.ca/). A volcano plot was utilized to compare group differences, employing fold change (FC) analysis and the Wilcoxon signed-rank test to identify statistically significant metabolites (p<0.05, |FC|>1.5). Heatmaps were generated to visualize variations in metabolite profiles. Pathway analysis was conducted based on significantly altered metabolites. Box plots were employed to illustrate mean values with standard deviations. Statistical analysis involved the use of the Mann-Whitney test and Pearson’s chi-square test, with a significance level set at p<0.05.

## Results

### Identification of the risk metabolite profile in PCa patients’ urine by UPLC-MS/MS

**Figure 1:**
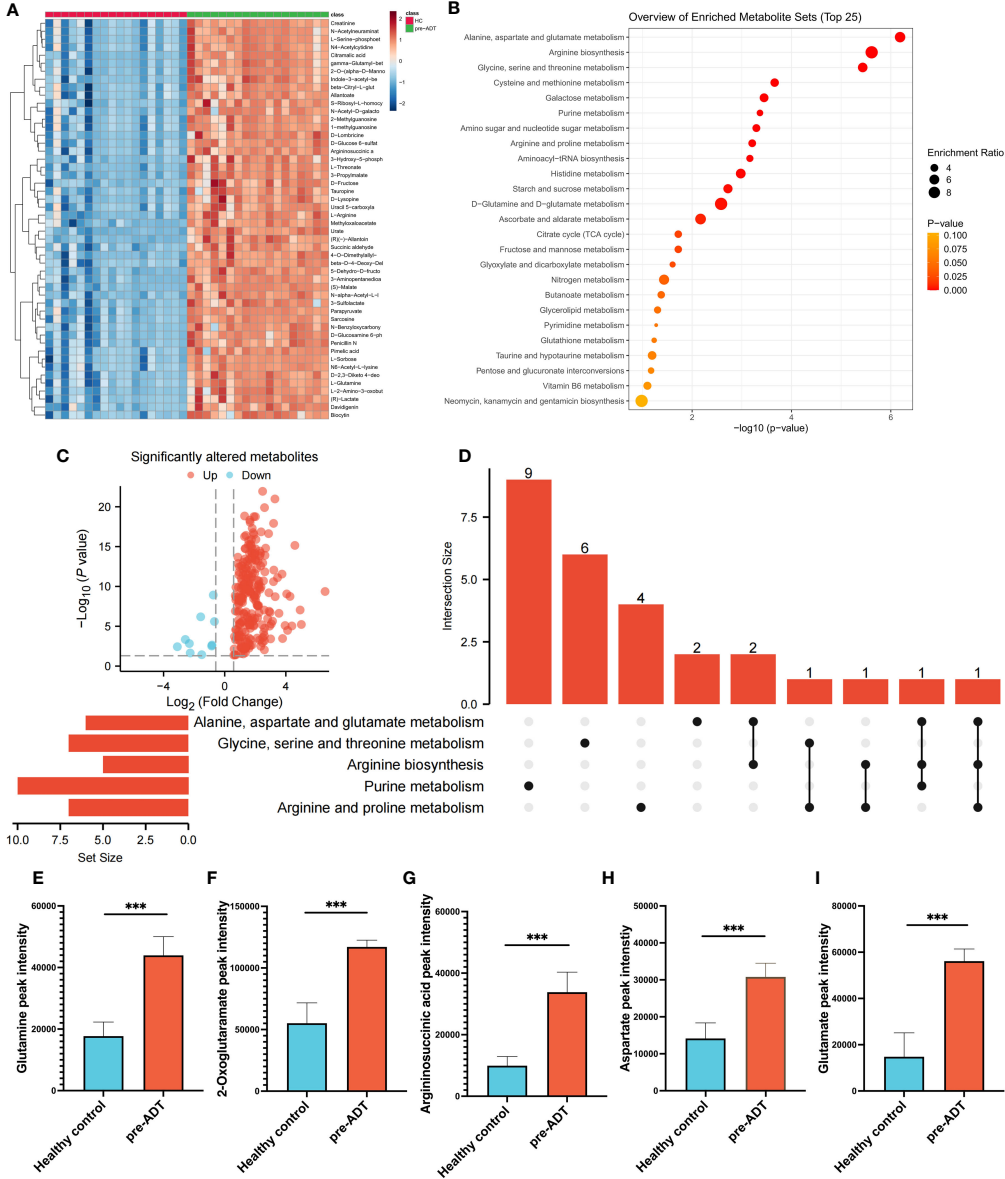
Dysregulation of metabolism between PCa and healthy samples. The landmark of the top 50 altered metabolites, red class for healthy samples and green class for PCa **(A)**. Pathway enrichment analysis based on all significantly altered metabolites **(B)**. The trend of all significantly altered metabolites, 223 upregulated and 10 downregulated **(C)**. Relationship among the top 5 enriched pathways **(D)**. Compared to healthy samples, the levels of glutamine **(E)**, 2-oxoglutaramate **(F)**, argininosuccinic acid **(G)**, aspartate **(H)** and glutamate **(I)** were significantly upregulated in the PCa group. (***p < 0.001).

Compared to healthy humans, the level of metabolites in treatment-naïve PCa patients was generally elevated (**Figure 1A**). Enrichment analysis based on differential metabolites revealed the typical metabolic reprogramming pathway in patients with prostate cancer (**Figure 1B**). The examination of a volcano plot revealed that 223 metabolites were dramatically elevated and 10 were markedly downregulated (**Figure 1C**). Using an UpSet plot, the hit number (the number of metabolites belonging to enriched particular pathways) and the overlapping relationship of the top 5 pathways were explored, aspartate and glutamate metabolism ranked first (10 hits), followed by arginine biosynthesis, glycine, serine, and threonine metabolism, cysteine and methionine metabolism, and galactose metabolism (**Figure 1D**). Five overlapping metabolites (glutamine, 2-oxoglutaramate, argininosuccinic acid, aspartate, and glutamate) were identified between aspartate and glutamate metabolism and arginine synthesis, the two most crucial processes (**Figures 1E–I**). To satisfy the increasing energy requirement, the elevated pathways and five metabolites might contribute to the initiation and progression of PCa. Determining the response of these metabolites to therapy is essential for clinical guidance.

### NaADT inhibits PCa metabolism but not the typical risk metabolite profile

**Figure 2:**
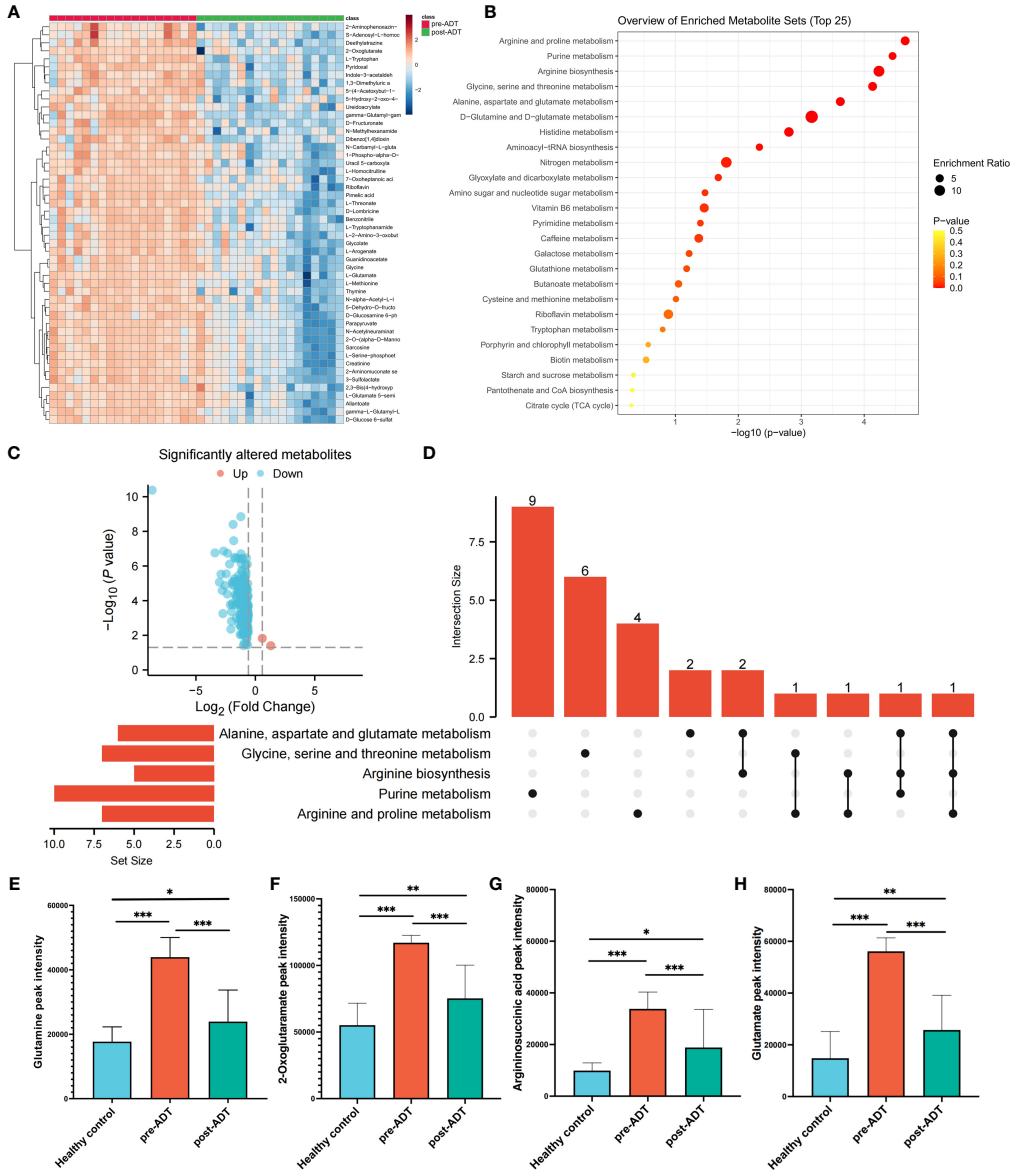
Metabolic alteration of PCa patients under naADT alone. The landmark of top 50 altered metabolites, red class for treatment-naïve group and green class for post-ADT group **(A)**. Pathway enrichment analysis based on all significantly altered metabolites **(B)**. The trend of all significantly altered metabolites, 2 upregulated and 162 downregulated **(C)**. Relationship among the top 5 enriched pathways **(D)**. The changes in glutamine **(E)**, 2-oxoglutaramate **(F)**, argininosuccinic acid **(G)** and glutamate **(H)** levels among the healthy group, treatment-naïve group and post-ADT group were visualized. (*p < 0.05; **p < 0.01; ***p < 0.001).

The heatmap based on the top 50 differential metabolites between treatment-naïve and post-naADT groups is shown in **Figure 2A**. Most of the metabolic expression was suppressed after naADT. The enriched pathways of these differential metabolites are shown in **Figure 2B**. The volcano plot demonstrated 164 significantly downregulated metabolites and 2 significantly elevated metabolites (**Figure 2C**). The UpSet plot was used to examine the hit count and overlapping relationships of the top 5 metabolic pathways, including arginine and proline metabolism (7 hits), purine metabolism (10 hits), arginine biosynthesis (5 hits), glycine, serine, and threonine metabolism (7 hits), and alanine, aspartate, and glutamate metabolism (7 hits) (**Figure 2D**). Four metabolites, glutamine, 2-oxoglutaramate, argininosuccinic acid, and glutamate, were significantly suppressed (p<0.05) by naADT in both the alanine, aspartate, and glutamate metabolism pathways and the arginine biosynthesis metabolism pathway (**Figures 2E–H**). Despite the fact that naADT considerably inhibited PCa metabolism, the alanine, aspartate, and glutamate metabolism pathways, which were the most active in PCa, were not the most inhibited by naADT. In addition, the post-naADT levels of glutamine, 2-oxoglutaramate, argininosuccinic acid, and glutamate remained considerably higher than those in healthy samples (**Figures 2E–H**).

### Patients with OM-HSPC respond less to naADT treatment than those with intermediate- or high-risk PCa

**Figure 3:**
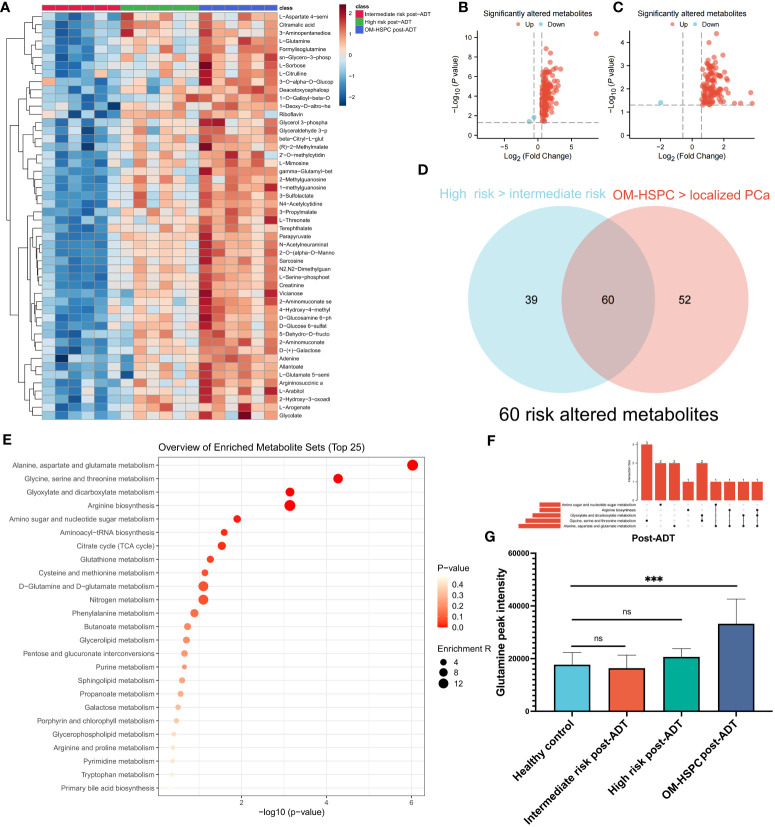
The difference in metabolites levels among intermediate-, high- and OM-HSPC post-ADT (pre-CIRT) patients. The landmark of the top 49 altered metabolites, red class for the intermediate-risk group, green class for the high-risk group and blue class for the OM-HSPC group **(A)**. 99 elevated metabolites were significantly altered between the high-risk and intermediate-risk groups **(B)**, and 112 upregulated and 2 downregulated metabolites were also revealed to be significantly altered between OM-HSPC and localized PCa **(C)**. The overlapping 60 metabolites were considered core risk metabolites **(D)**. Pathway enrichment analysis based on 60 core risk metabolites **(E)**. Relationship among the top 5 enriched pathways **(F)**. The changes in glutamine levels among stages post-ADT were visualized **(G)**. (***p < 0.001). ns, no significance.

The patients were separated into three groups, intermediate-risk and high-risk, and OM-HSPC, to examine the response to ADT at various stages. The heatmap (**Figure 3A**) with the top 50 metabolites revealed distinct differences between the three groups. Notably, the OM-HSPC group had significantly higher metabolic activity than the localized stages. Volcano plots (**Figure 3B**) identified 99 metabolites that were significantly elevated in the comparison of the high-risk and intermediate-risk groups, Similarly, 112 upregulated and 2 downregulated metabolites were also significantly altered in OM-HSPC compared to localized stages (intermediate- and high-risk) PCa (**Figure 3C**). From the two groups of upregulated metabolites, 60 overlapping metabolites (**Figure 3D**) were identified (**Supplementary Table 1**), which could be considered key risk metabolites associated with staging under ADT. Pathway enrichment analysis and UpSet plots revealed that the 60 identified metabolites were associated with multiple metabolic pathways, including alanine, aspartate, and glutamine metabolism (6 hits); Glycine, serine, and threonine metabolism (5 hits); Glyoxylate and dicarboxylate metabolism (4 hits); Arginine biosynthesis (3 hits); Amino sugar and nucleotide sugar metabolism (3 hits) (**Figures 3E, F**). Glutamine was discovered to be enriched in aspartate and glutamine metabolism as well as arginine biosynthesis. This finding suggested that glutamine levels differed significantly between post-naADT stages. Moreover, the glutamine levels of the intermediate- and high-risk groups treated with naADT were not substantially greater than those of the healthy samples, while glutamine levels were still considerably greater in the OM-HSPC group than in healthy samples or those with localized PCa (**Figure 3G**).

These findings highlight that glutamine plays a pivotal role in the initiation and progression of PCa. Furthermore, naADT appears to be less effective in reducing glutamine levels in OM-HSPC, compared to its impact on localized PCa, to the levels observed in healthy individuals. Consequently, the elevated glutamine levels in OM-HSPC suggest that relying solely on systemic naADT treatment may be inadequate in managing PCa, which could lead to suboptimal patient outcomes.

### Gene expression confirms the correlation of glutamine metabolism with PCa development and BCR

**Figure 4:**
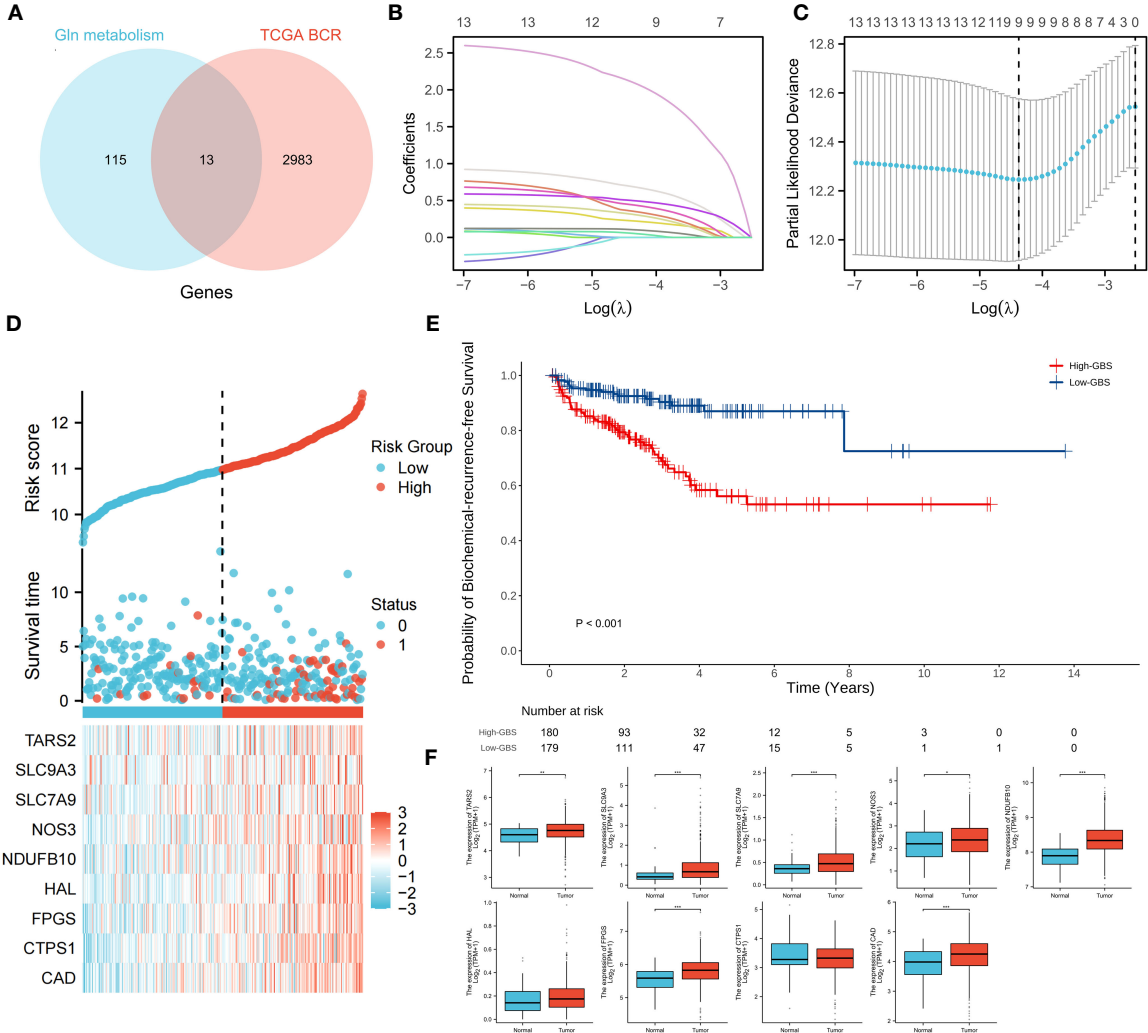
Relationship between glutamine metabolism-related genes and BCR of PCa. 13 overlapping genes from glutamine metabolism-related genes and BCR risk genes **(A)**. The regression process of the LASSO algorithm **(B, C)**. Relationship between the expression of 9 genes and clinical status **(D)**. BCR-free survival analysis based on GBS **(E)**. Expression of 9 genes between PCa and normal samples **(F)**. (*p < 0.05; **p < 0.01; ***p < 0.001).

The genes associated with glutamine metabolism from MSigDB and the BCR-related genes identified by univariate Cox regression (p<0.05, HR>1) from TCGA shared 13 genes, indicating that glutamine metabolism may play a risk factor in PCa BCR (**Figure 4A**). Based on TCGA data, a LASSO analysis with the glmnet package revealed 9 of the 13 genes (**Figures 4B, C**). Samples were categorized into low- and high-GBS groups based on the glutamine and BCR-related gene score (GBS) generated from the levels of expression of the 9 genes. **Figure 4D** shows the gene expression pattern for these groups. According to the findings, samples with relatively high GBS had considerably shorter BCR-free times (P< 0.001) (**Figure 4E**).

For each gene in PCa, a comparison of paracancerous and tumor tissue was carried out to learn more about the oncogenic role of these genes. TARS2, SLC9A, SLC7A9, NOS3, NDUFB10, FPGS, and CAD all showed significant changes (P< 0.05) (**Figure 4F**), showing that glutamine metabolism may play a role in the development and recurrence of PCa. Using TCGA PCa baseline data, the relationship between GBS and clinical PCa characteristics was also explored (**Table 3**). The findings showed that patients in the high-GBS group had significantly higher T stages and Gleason scores (P < 0.001).

**Table 3:** Patients’ clinical characteristics from TCGA database.

Characteristics	Low_GBS	High_GBS	*p* value
n	179	180	
Age, median (IQR)	61 (55.5, 66)	62 (57.75, 66)	0.314
T stage, n (%)			< 0.001
T2a	2 (1.1%)	3 (1.7%)	
T2b	3 (1.7%)	5 (2.8%)	
T2c	68 (38%)	40 (22.2%)	
T3a	70 (39.1%)	52 (28.9%)	
T3b	33 (18.4%)	75 (41.7%)	
T4	3 (1.7%)	5 (2.8%)	
Gleason score, n (%)			< 0.001
<=7	115 (64.2%)	77 (42.8%)	
>7	64 (35.8%)	103 (57.2%)	

GBS: glutamine metabolism genes and biochemical recurrence related scores.

The results indicate a notable correlation between heightened glutamine-related metabolism and advanced stages of PCa, as well as a reduced BCR-free survival duration. As such, reducing glutamine levels in OM-HSPC patients might enhance their prospects for improved local control.

### Glutamine and other main risk metabolites were considerably decreased in OM-HSPC patients treated with naADT followed by CIRT

**Figure 5:**
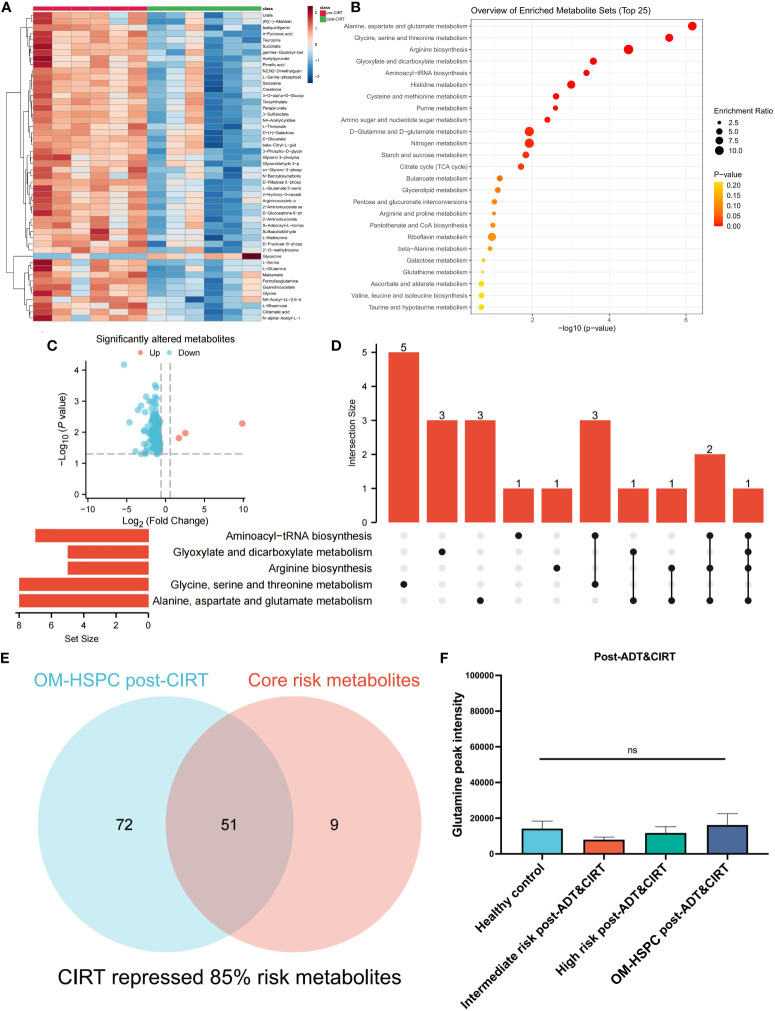
Metabolic alteration after CIRT combined with naADT for OM-HSPC. The landmark of the top 50 altered metabolites, red class for pre-CIRT, green class for post-CIRT **(A)**. Pathway enrichment analysis based on significantly altered metabolites **(B)**. Three upregulated and 120 downregulated metabolites were revealed to be significantly altered after CIRT for OM-HSPC **(C)**. Relationship among the top 5 enriched pathways **(D)**. 85% of risk metabolites were downregulated by CIRT **(E)**. The levels of glutamine after CIRT **(F)**.

The metabolic profiles of OM-HSPC patients before and after CIRT are depicted in **Figure 5A**. The enrichment analysis uncovered significantly downregulated metabolic pathways (**Figure 5B**). The volcano plot revealed that 3 metabolites were considerably elevated and 120 were significantly downregulated (**Figure 5C**). The UpSet analysis found the top 5 altered metabolic pathways to be alanine, aspartate, and glutamate metabolism (6 hits); Glycine, serine, and threonine metabolism (5 hits); Arginine biosynthesis (5 hits); Glyoxylate and dicarboxylate metabolism (4 hits) (**Figure 5D**). The Venn diagram represented the association between the 123 differential metabolites and the 60 risk metabolites identified in prior investigations (**Figure 5E**). After CIRT, glutamine levels in all three groups were reduced to healthy levels (p>0.05) (**Figure 5F**).

**Figure 6:**
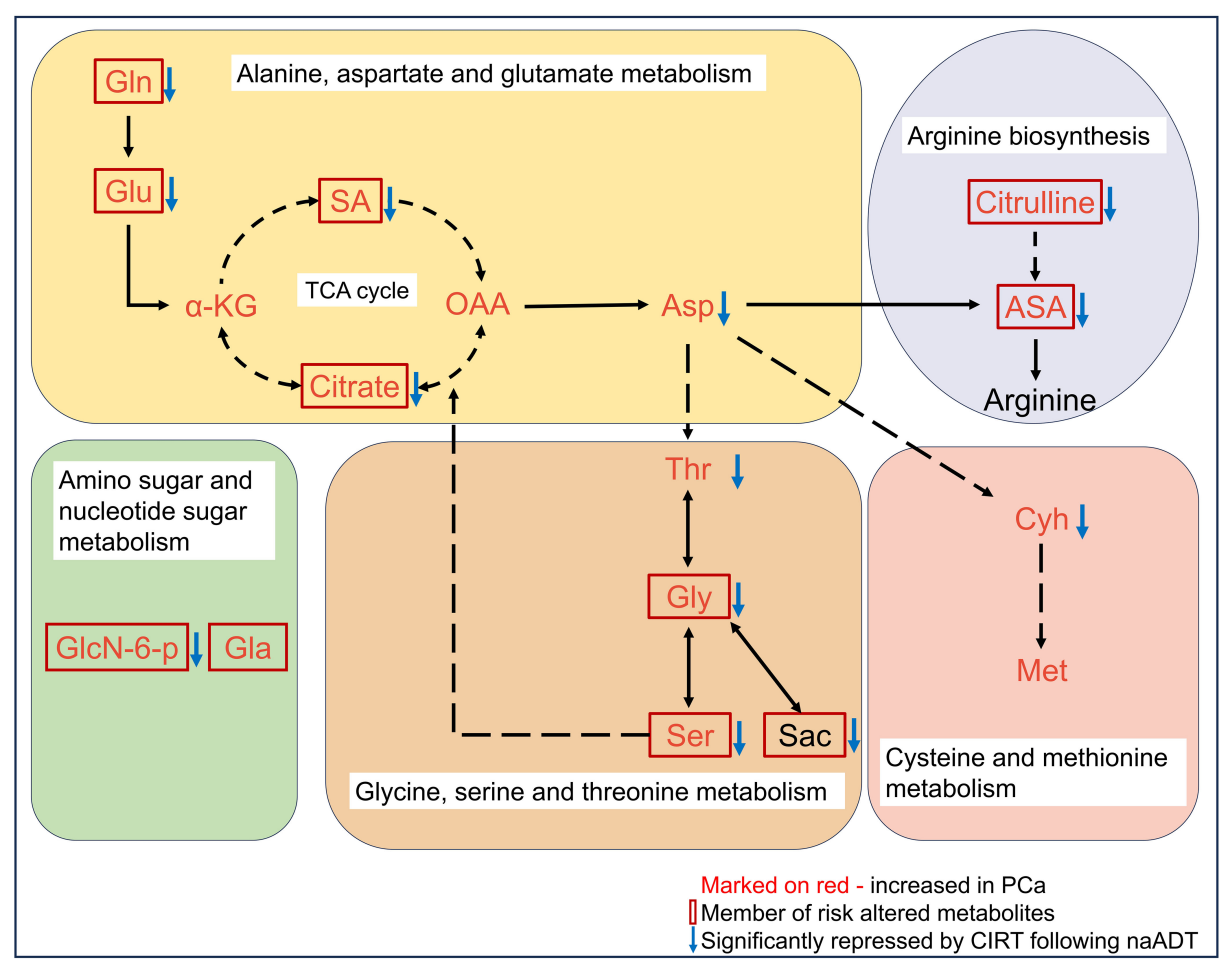
Potential impacts of CIRT combined naADT on metabolic change. Gln, glutamine; Glu, glutamate; α-KG, 2-Keto-glutaramic acid; SA, succinic acid; OAA, oxoglutaric acid; Asp, aspartate; ASA, argininosuccinic acid; GlcN-6-p, N-Acetyl-D-glucosamine 6-phosphate; Gla, galactose; Thr, threonine; Gly, glycine; Ser, serine; sar, sarcosine; Cyh, cystathionine; Met, methionine.

85% (51 out of 60) risk metabolites were considerably decreased in patients treated with naADT followed by CIRT. In addition, the glutamine levels of OM-HSPC patients were reduced to a level comparable to that of healthy individuals. Based on present conclusions, we depicted the changes among core risk metabolites influenced by treatments in **Figure 6**.

### The suppression of glutamine by CIRT in PCa tumor tissue in a tumor-xenografted mouse model

**Figure 7:**
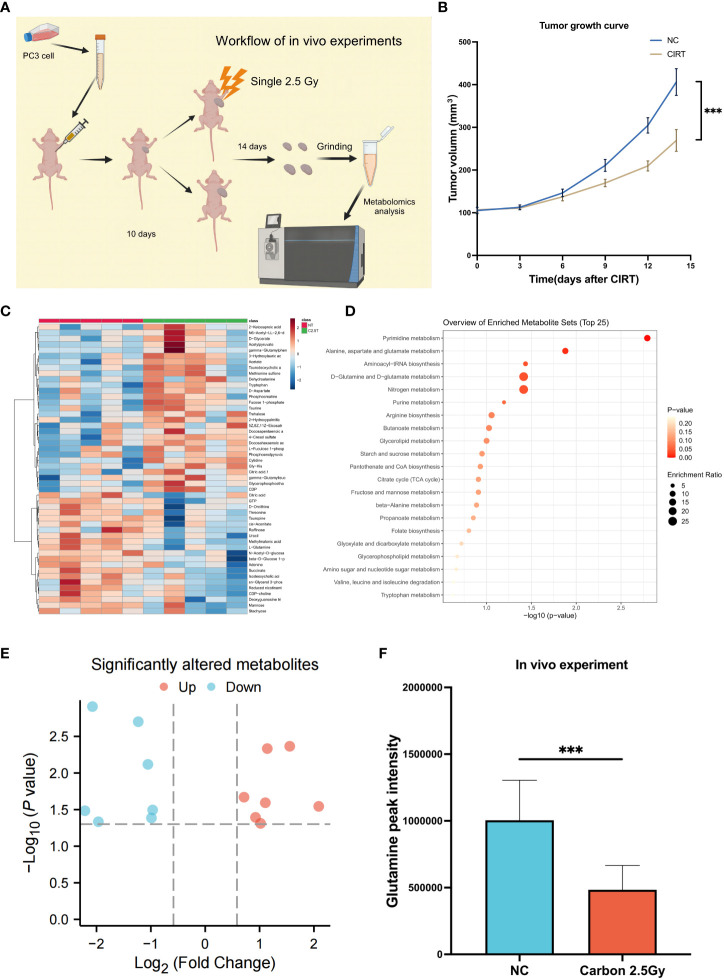
Validation experiments of mouse model *in vivo*. Workflow of the *in vivo* experiment **(A)**. Tumor growth curves of the two groups **(B)**. The landmark of the top 50 altered metabolites, red class for the NC group and green class for the irradiated group, single 2.5 Gy **(C)**. Pathway enrichment analysis based on all significantly altered metabolites **(D)**. The trend of all significantly altered metabolites, 7 upregulated and 7 downregulated **(E)**. Compared to the NC group, the level of glutamine was significantly downregulated after CIRT **(F)**. (*p < 0.05; **p < 0.01; ***p < 0.001).

Due to the impossibility of obtaining tissue samples from PCa patients treated with CIRT, an experiment on mouth was conducted to confirm the inhibitory effects of CIRT on glutamine levels in tumor tissue. The mice were divided into two independent groups: normal control (NC) and CIRT. The CIRT group was irradiated with a single fraction of 2.5 Gy carbon ion. NC was the nontreatment control group. Tumor size was measured every three days during this time period, and tumor samples were collected 14 days after irradiation, (**Figure 7A**). It was evident that CIRT dramatically inhibited tumor growth compared to the NC group (**Figure 7B**). Metabolomic analysis of tumor tissue indicated distinct metabolites in the two groups (**Figure 7C**), with seven downregulated and seven upregulated (**Figure 7E**). Enrichment analysis found the differential pathways (**Figure 7D**), which revealed a substantial decrease in glutamine levels in the CIRT group compared to the NC group, with a p value less than 0.001 (**Figure 7F**). This result showed the ability of CIRT to inhibit glutamine metabolism in PCa tissue.

### Glutamine deprivation enhances the inhibitory effects of CIRT on tumor migration and invasion

**Figure 8:**
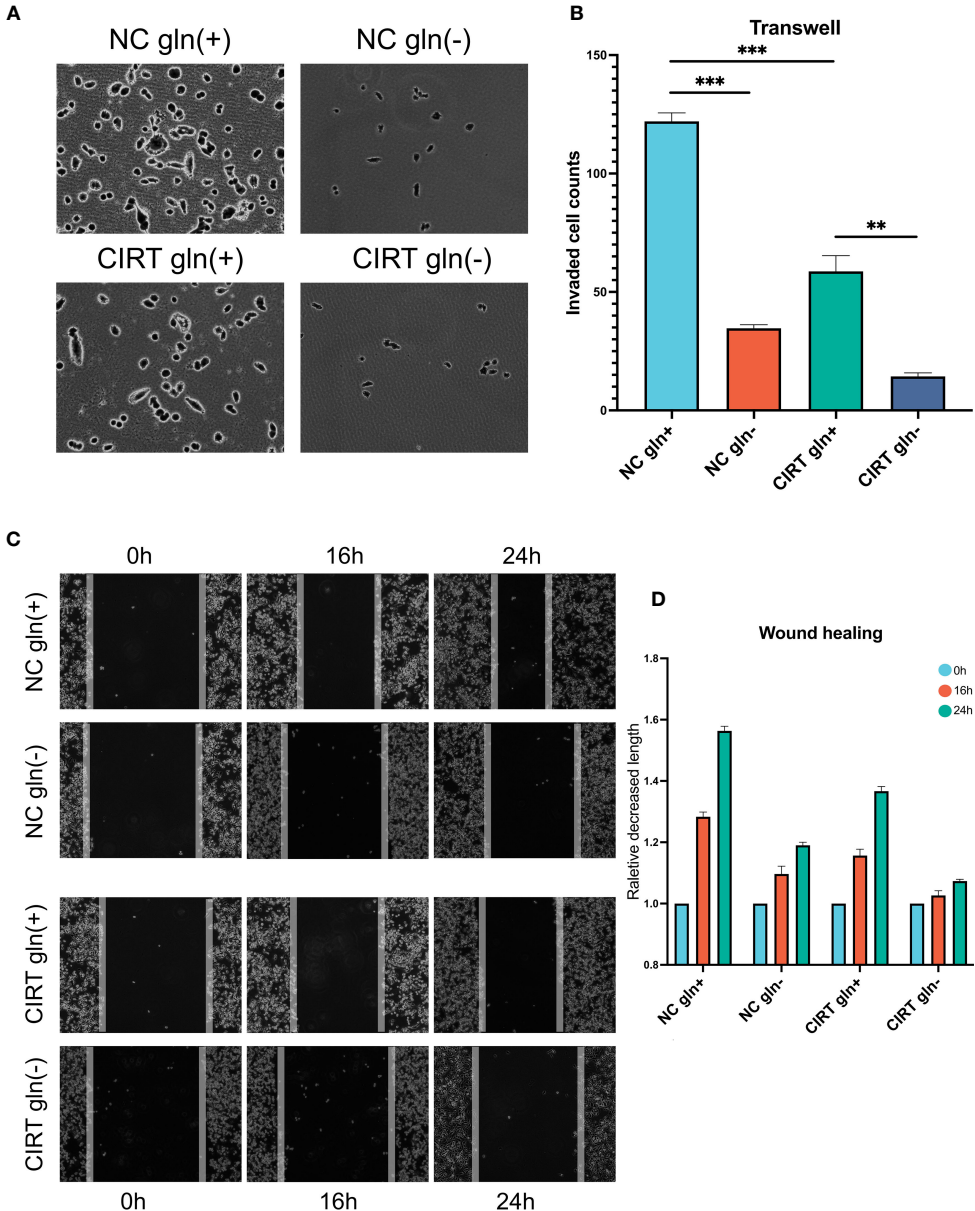
Validation and exploration with *in vitro* cell experiments. After 48 h, compared to the NC gln(+) group, both glutamine deprivation and CIRT significantly impaired the invasive ability of PCa cells, and the CIRT with glutamine deprivation group showed the lowest invasive cell counts **(A, B)** (40X). Compared to the NC gln(+) group, both glutamine deprivation and CIRT significantly impaired the migrative ability of PCa cells, and the CIRT with glutamine deprivation group showed the longest remaining distance after 24 h **(C, D)** (40X).

Given that glutamine plays a crucial role in the progression of PCa, we employed PC3 cells as a prostate model to determine whether glutamine deprivation could enhance the inhibitory effect of CIRT on PCa invasion and migration. In Transwell tests, 48 hours after 2 Gy carbon ion irradiation, less cells retained the capacity to infiltrate Matrigel to the bottom of the insert (**Figures 8A, B**). When glutamine was removed from the cell media, cell invasion was drastically reduced. In addition, the cell migratory ability was also decreased by the single dose of 2 Gy carbon ion irradiation in wound healing assay. When glutamine was eliminated from the cell media, cell migration was further remarkably impaired. These results preliminarily indicated that glutamine deprivation could significantly enhance the capacity of CIRT to inhibit tumor metastasis.

## Discussion

Carbon ion irradiation has been gradually regarded as an effective treatment strategy for localized prostate cancer because of its lower side effects and higher radiobiological benefits (RBE) (16, 17). Several studies found that CIRT improved the treatment outcome in patients with localized prostate cancer although no trials concerning CIRT have been performed in the definitive setting of OM-HSPC. Exploration of the underlying mechanism will add benefit to improve the clinical outcome of OM-HSPC.

The present study is the first to evaluate the unique radiobiological effect of CIRT from a metabolomics perspective for OM-HSPC. For this phase before widely metastatic PCa, this study provides a deeper understanding of initiation- and progression-related metabolism and the radiometabolic effects of CIRT. The results elucidate the correlation between risk metabolites, specifically glutamine metabolism, and PCa malignancy and preliminarily explain the synergistic metabolic remolding effects of naADT and CIRT.

Multiple studies have consistently observed the following in urinary metabolomics of prostate cancer: citrate, glutamine, glutamate, glycine, tyrosine, 2,6-dimethyl-7-octen-2-ol, citrulline, histidine, succinate, kynurenic acid, taurine, leucine, and pyruvate (18). In this study, we compared the metabolic profiles of PCa patients and healthy individuals and discovered that the 223 differential metabolites were primarily enriched in aspartate and glutamate metabolism (10 hits); Arginine biosynthesis; Glycine, serine, and threonine metabolism; Cysteine and methionine metabolism; Galactose metabolism (**Figure 1D**). Five overlapping metabolites, glutamine, 2-oxoglutaramate, argininosuccinic acid, aspartate, and glutamate, were deemed to have a strong connection with prostate cancer (**Figures 1E–I**). Risk urinary metabolites such as glutamate, glutamine, were similar to those reported in prior metabolomics studies (18). In our research, we discovered novel arginine metabolites that were also considered risk metabolites of PCa.

Prior research has similarly indicated a correlation between glutamine levels and the initiation and progression of PCa (19). However, the nuances of how glutamine metabolism changes under ADT in human samples remain largely unexplored. For decades, ADT has stood as the primary treatment modality for PCa. Previous studies suggest that ADT suppresses steroid synthesis and ketogenesis in the plasma, and amplifies bile acids and their associated metabolites (20, 21). Yet, in the current study, ADT demonstrated a limited ability to diminish PCa-associated risk metabolites, especially glutamine (as shown in **Figure 2A**). Following naADT treatment, there was a significant reduction in glutamine levels, but these levels still exceeded those found in healthy individuals (as shown in **Figures 2F–I**). Notably, OM-HSPC patients exhibited the highest concentration of risk metabolites among all groups, with glutamine being particularly elevated.

Further, an analysis of TCGA PCa RNA-seq data revealed an association between increased expression of glutamine-related metabolism genes and both higher-stage populations and reduced BCR-free survival. Confirming this, other independent studies also underscored the prognostic importance of genes related to glutamine metabolism in PCa (22, 23). The transporters responsible for alanine, serine, cysteine, and glutamine uptake (known as ASCT2 or SLC1A5) are pivotal in glutamine metabolism (24, 25). The expression of ASCT2 intensifies in patients with recurrent PCa post-ADT (26), ASCT2 is notably overexpressed in cancerous prostate cells and inhibiting ASCT2 could impair the tumor cell growth and development of metastases in PCa xenografts models (26). This could explain the elevated glutamine levels in OM-HSPC following naADT, potentially contributing to less favorable outcomes. In conclusion, relying solely on ADT to treat OM-HSPC patients might be insufficient in curtailing the risk metabolites.

In our previous research, we found that CIRT markedly suppressed urine metabolites in most localized PCa patients (8). In this current study, we pinpointed metabolites associated with the initiation and progression of PCa. For OM-HSPC, an elevated urinary metabolic level, especially in terms of glutamine-related metabolism, may pose a prognostic risk when relying solely on ADT. Intriguingly, when naADT treatment was combined with CIRT, 85% (51/60) of the risk metabolites were reduced. Notably, the urinary glutamine levels in OM-HSPC patients dropped to, or even below, levels observed in both localized cases and healthy samples post-combination treatment (as shown in **Figure 5G**). These findings shed light on the mechanism behind the enhanced therapeutic outcomes of combined naADT-CIRT. Additionally, our study underscores the distinct biological impacts of CIRT. These insights also enhance our understanding of the metabolic traits of advanced PCa. Additionally, for OM-HSPC CIRT might evoke the systemic antitumor immune responses (27, 28) by facilitating the immunogenic cell death and remolding the immune microenvironment, especially through the metabolism remolding.

Another compelling dimension of this study is the heightened efficacy of naADT-CIRT therapy under glutamine restriction. When glutamine was excluded from the cell medium, CIRT’s inhibitory impact on cell invasion and migration intensified, underscoring the significance of glutamine deprivation in augmenting CIRT’s effectiveness. While prior studies have discussed glutamine dependency in PCa, this presents the inaugural evidence that glutamine deprivation can potentiate the effects of CIRT.

## Conclusion

Our study pinpointed a set of core risk metabolites, with urinary glutamine levels correlating with PCa’s initiation, progression, and response to ADT, positioning it as a potential biomarker and therapeutic target. Furthermore, while naADT and CIRT each have their unique metabolic impacts, their combined effect effectively curtails most core risk metabolites in OM-HSPC, especially glutamine. This provides a mechanistic foundation for their combined therapeutic success in OM-HSPC. Additionally, we unveiled, for the first time, that depleting glutamine amplifies CIRT’s anti-invasion and anti-migration effects on PCa.

## Data availability statement

Bioinformatics datasets and metabolomics data presented in this study can be found in online repositories. The datasets used and/or analyzed during experiments are available from the corresponding author upon reasonable request.

## Ethics statement

The studies involving humans were approved by Ethics Committee of the Shanghai Proton and Heavy Ion Center, Fudan University Cancer Hospital. The studies were conducted in accordance with the local legislation and institutional requirements. The participants provided their written informed consent to participate in this study. The animal study was approved by Shanghai Proton and Heavy Ion Center approved the use of mice for experiments. The study was conducted in accordance with the local legislation and institutional requirements.

## Author contributions

ZZ: Conceptualization, Data curation, Formal Analysis, Investigation, Methodology, Software, Writing – original draft, Writing – review & editing. YP: Methodology, Resources, Software, Writing – review & editing. WH: Writing – review & editing. YX: Writing – review & editing. RN: Resources, Supervision, Writing – review & editing. XG: Resources, Supervision, Validation, Writing – review & editing. YS: Formal Analysis, Funding acquisition, Methodology, Project administration, Software, Writing – review & editing. QZ: Conceptualization, Funding acquisition, Resources, Supervision, Validation, Writing – review & editing.
